# Association of affect with vertical position in L1 but not in L2 in unbalanced bilinguals

**DOI:** 10.3389/fpsyg.2015.00693

**Published:** 2015-05-27

**Authors:** Degao Li, Haitao Liu, Bosen Ma

**Affiliations:** Department of Language and Translation, School of International Studies, Zhejiang UniversityHangzhou, China

**Keywords:** unbalanced bilinguals, metaphorical association, affective words, second language

## Abstract

After judging the valence of the positive (e.g., *happy*) and the negative words (e.g., *sad*), the participants' response to the letter (*q* or *p*) was faster and slower, respectively, when the letter appeared at the upper end than at the lower end of the screen in Meier and Robinson's (2004) second experiment. To compare this metaphorical association of affect with vertical position in Chinese-English bilinguals' first language (L1) and second language (L2) (language), we conducted four experiments in an affective priming task. The targets were one set of positive or negative words (valence), which were shown vertically above or below the center of the screen (position). The primes, presented at the center of the screen, were affective words that were semantically related to the targets, affective words that were not semantically related to the targets, affective icon-pictures, and neutral strings in Experiment 1–4, respectively. In judging the targets' valence, the participants showed different patterns of interactions between language, valence, and position in reaction times across the experiments. We concluded that metaphorical association between affect and vertical position works in L1 but not in L2 for unbalanced bilinguals.

## Introduction

Humans tend to judge the concept of “highness” as good and “lowness” as bad (Crawford, 2009). We posed the question: Does this metaphorical association between affect and vertical position work in the same way in a second language (L2) as it does in a first language (L1) in unbalanced bilinguals? Inspired by previous studies (e.g., Duyck and Brysbaert, 2004; Bialystok et al., 2006) we refer to those as unbalanced bilinguals who learn an L2 mainly in classroom settings after the age of seven and achieve a far lower level of proficiency in L2 than in L1. An answer to the above-mentioned question will help enrich our understanding of bilinguals' representations for affective words (e.g., *happy* and *sad*) in L2.

Two early theories on bilinguals' semantic representations are the word association model and the concept mediation model (Potter et al., 1984). The former assumes that L2 words cannot be understood without being translated into L1 words. The latter suggests that the L1 and L2 lexicons are independently connected to conceptual memory. Kroll and Stewart (1994) merged these two theories and proposed the revised hierarchical model (RHM), providing a useful framework for research into the relationship between bilinguals' mental lexicons and semantic representations. In the RHM, the three memory stores are connected by links of different strengths. The link between the L1 lexical store and the conceptual store is strong while the link between the L2 lexical store and the conceptual store is weak. Bilinguals who have a low level of proficiency in L2 can understand the meanings of L2 words by associating with the corresponding L1 translation equivalents. Their strength of link between the L2 lexical store and the conceptual store increases as their L2 proficiency rises.

Another important theory is the distribution conceptual feature model (De Groot, 1992), in which lexical nodes are associated with a distributed set of conceptual features and the degree of overlap of conceptual features between words in L1 and L2 depends on what is represented. That is, more concrete words (e.g., *apple*) are different from more abstract words (e.g., *fruit*) in how the conceptual representations are shared by L1 and L2. Basing their findings on words with multiple meanings, Finkbeiner et al. (2004) proposed the sense model, which assumes that each sense of a word constitutes a distinct mental representation and that existence of a representational asymmetry between related words is possible. Given the semantic and the emotional aspects of affective words, however, the existing theories may not be promising enough to predict bilinguals' representations for this subset of words in L2.

Affective words are words that can be evaluated as positive or negative in meaning (Clore et al., 1987). Being affective is a matter of degree (Thayer, 1989), and some words are more positive or negative than other words. Different from words (e.g., *table*, *universe*, and *theory*) that are comparatively neutral in valence, affective words are likely to provoke one's emotional states. Indeed, researchers have recognized that affective words have two key components, valence (pleasantness at encoding affective words that varies from strongly negative to neutral to strongly positive) and arousal (the extent of calmness or excitation) (Kensinger, 2009), which have significant influences on monolinguals' cognitive activities (see Ferré et al., 2011, for review). For example, automatic processing of affective information can be measured not only in affective tasks (e.g., tasks in which valence judgments are required on the targets) but also in a priming task of semantic categorization (Spruyt et al., 2007). Although largely moderated by age of L2 acquisition in bilinguals (Altarriba, 2008), affective words are intuitively more arousing in emotion in L1 than in L2 (Dewaele, 2004). However, empirical studies seem to have yielded inconsistent results on the influence of valence of affective words in L1 and L2 on bilinguals' performance in cognitive tasks.

For example, English-Spanish bilinguals as well as Spanish-English bilinguals rated the pronunciation and emotionality and then recalled the words, both groups of bilinguals had higher accuracies for affective words than for neutral words in L1 but not in L2 (Anooshian and Hertel, 1994). Ayçiçegi and Harris (2004) extended the research of Anooshian and Hertel (1994) and found that Turkish-English bilinguals had better memories for affective words than for neutral words in both L1 and L2. After a series of tasks on affective words, Turkish–English bilinguals had a similar pattern of free-recall performance in L1 and L2 (Ayçiçeği-Dinn and Caldwell-Harris, 2009). Similarly Ferré et al. (2010, 2012) indicated that proficient bilinguals had the same magnitude of superiority in recalling affective over neutral words in L2 as they had in L1. Ignoring meaning and reporting the color of visually presented words in a modified Stroop task, Finnish-English bilinguals appeared equally able to make responses to affective stimuli in L1 and L2 (Eilola et al., 2007). In classifying words' valence or color, both Spanish-English and English-Spanish bilinguals showed a similar pattern of performance in L1 and L2 in an affective Simon task (Altarriba and Basnight-Brown, 2011). Harris et al. (2003) asked proficient Turkish-English bilinguals to rate how pleasant certain positive and negative words were and recorded their skin conductance responses (SCRs). They found no significant differences in the participants' responses between languages. However, Harris et al. (2006) indicated differences between early and late bilinguals' SCR responses to emotional words. Furthermore, Eilola and Havelka (2010) required native and non-native speakers of English to complete an affective Stroop task and measured their SCRs. The native speakers responded with higher SCRs to the negative than to the neutral or the positive words, but the non-native speakers did not show such a pattern of changes. In a different way, Opitz and Degner (2012) measured French-German and German-French bilinguals' event related potentials in word reading and showed that affective words might be processed with less immediacy in L2 than in L1. Altarriba and Canary (2004) suggested that the priming effect was weaker for Spanish-English bilinguals than for English monolinguals in Fazio's et al. (1986) affective priming task. Degner et al. (2012) did a similar study on German-French and French-German bilinguals and affective priming effects were found in L1 but not in L2 for those who did not have high levels of language immersion or frequency use of L2.

Obviously, differences in bilinguals' age and the context of acquisition of L2 as well as their proficiency in L2 contributed to the result variety in the aforementioned studies. Studies are needed to provide more evidence on unbalanced bilinguals' representations for affective words in L2.

## The present study

Children develop their thinking ability basing on what they see, what they hear, etc. (Piaget and Inhelder, 1969), and their representations for affective words cannot be free from their sensorimotor experiences. Similarly, Lakoff and Johnson (1999) argued that “conceptual thought is almost always grounded in physical metaphor in a manner that is relatively implicit … our ability to represent abstract experiences (e.g., affect) is, in part, built on our ability to represent perceptual experiences (e.g., brightness, vertical position, distance)” (Meier and Robinson, 2009, pp. 240–241). Apart from these theoretical arguments, empirical studies emerge in support of metaphorical associations between perception and affect. For example, participants were likely to rate football teams in dark uniforms as more malevolent than those in bright uniforms (Frank and Gilovich, 1988). Participants performed better in evaluating positive words that were presented in a white font than in a black font (Meier et al., 2004) and in evaluating positive words that were presented in a big font than in a small font (Meier et al., 2008).

Most inspiring to us, however, was Meier and Robinson's (2004) Experiment 2. Immediately after deciding the valence of an affective word that was shown in a trial at the center of the screen, participants pressed a key on the keyboard corresponding to the letter (*q* or *p*) that appeared in the top or in the bottom of the screen. Their responses were faster to the letters presented at the upper end of the screen than to those at the lower end for the positive words and were faster to the letters in the lower than to those in the upper position for the negative words. “Representations of goodness activate higher areas of visual space, whereas representations of badness activate lower areas of visual space” (Meier and Robinson, 2004, p. 247).

Because of the graphic difference between Chinese and alphabetic languages, we were unable to directly adopt Meier and Robinson's (2004) methodology. Since Fazio's et al. (1986) affective priming task appears to be a nice instrument to reveal differences in bilinguals' performance on affective words in L1 and L2 (Altarriba and Canary, 2004; Degner et al., 2012), we combined it with Meier and Robinson's (2004) task and created a modified version of affective priming task (MAPT) in order to compare unbalanced bilinguals' association between valence and vertical position in L1 and L2.

In Fazio's et al. (1986) affective priming task two affective words are sequentially presented in a trial. Participants' semantic judgment on the target is fostered if the target and the prime are congruent and is not fostered if the target and the prime are incongruent in valence. Considering unbalanced bilinguals' limited vocabulary in L2, we did not take valence congruency between the prime and the target as a variable. Thus, in a trial in MAPT, the prime would be shown at the center of the screen, but the target of the same valence as the prime would be presented vertically above or below the center of the screen in a trial.

Thus, we arrived at the following hypothesis.

*If affect was associated with vertical position for unbalanced bilinguals in L2, then their decisions on positive targets would be fostered more at the upper than at the lower position and their decisions on negative targets would be fostered more at the lower than at the upper position on the screen in MAPT*.

## Experiments

Given that semantic processing parallels affective processing in participants' automatic perception of the affective primes in an affective priming task (Pavlenko, 2012), we conducted a series of four experiments to test the above-mentioned hypothesis. The target was an affective word in a trial and the prime was (1) an affective word that was semantically related to the target in Experiment 1, (2) an affective word that was not semantically related to the target in Experiment 2, (3) an affective icon-picture in Experiment 3, and (4) a neutral, meaningless string of symbols in Experiment 4. Different amounts of priming effect were expected across these experiments. When the prime was a word and was semantically related to the target, both semantic and affective priming effect was expected; when the prime was a word but was not semantically related to the target, only affective priming effect was expected; when the prime was an icon-picture instead of an affective word, the same amount of affective priming effect was expected when the target was in L1 and L2; when the prime was a meaningless, neutral string, no priming effect was expected at all.

### Method

The design formed a 2 (language: Chinese or English) × 2 (valence: positive or negative) × 2 (position: upper or lower) repeated factorial in every experiment, and the dependent variables were error rates and reaction times.

#### Participants

Sixty college students (32 females) (*M* = 20.1 years, age range: 18.6–20.7 years), majoring in engineering specialties, were recruited on campus by means of a flyer advertisement. From a brief questionnaire we learned that the students had begun learning English in classroom settings at or after the age of 10 and were of the same level of proficiency in English as indicated in the placement test at the beginning of the first semester. They also attended a 60-item version of the *Quick Placement Test* issued by Oxford University Press and achieved an average score of 43.6 ± 10.9 (*M* ± *SD*). According to the test criterion, a student's proficiency is of an intermediate, an advanced, and a proficient level if he or she achieves a score between 40 and 47, between 48 and 54, and between 55 and 60, respectively. The participants were divided into four equal groups (eight female students in every group) and the four groups were not significantly different from one another in their scores in the placement test, *F*_(3, 56)_ = 0.085, *MSE* = 4.44, *p* = 0.968. Participant groups 1–4 would attend Experiments 1–4, respectively.

#### Materials

With reference to Altarriba and Canary (2004), thirteen pairs of positive and 13 pairs of negative words in Chinese and the same number of affective words in English (see Appendix I) were determined, which were used as the critical materials in Experiment 1. Twenty-five college students evaluated the valence of each word on a seven-point scale. Similarly, a second, third, and forth group of 25 college students evaluated the arousal, the familiarity, and the concreteness of each word, respectively. The evaluation scores were not significantly different between the primes and the targets in familiarity, concreteness, valence, or arousal in L1 [*t*_(25)_ = 0.277, *p* = 0.789; *t*_(25)_ = 1.557, *p* = 0.132; *t*_(25)_ = 0.706, *p* = 0.487; *t*_(25)_ = 0.771, *p* = 0. 448] or L2 [*t*_(25)_ = 0.299, *p* = 0.767; *t*_(25)_ = 0.890, *p* = 0.382; *t*_(25)_ = 0.161, *p* = 0.873; *t*_(25)_ = 0.303, *p* = 0.763]. The positive primes (6.12 ± 0.58; 6.11 ± 0.43) and targets (6.23 ± 0.55; 6.20 ± 0.58) were significantly higher than the negative primes (1.95 ± 0.61; 2.09 ± 0.59) and targets (1.68 ± 0.33; 1.96 ± 0.52), respectively, in valence in L1 [*t*_(24)_ = 17.748, *p* = 0.000, *d* = 7.006^1^; *t*_(24)_ = 25.675, *p* = 0.000, *d* = 10.032] and L2 [*t*_(24)_ = 19.858, *p* = 0.000, *d* = 7.787; *t*_(24)_ = 19.646, *p* = 0.000, *d* = 7.698]. The positive primes and targets were not significantly different from the negative primes and targets, respectively, in familiarity [*t*_(24)_ = 0.982, *p* = 0.367; *t*_(24)_ = 0.423, *p* = 0.621], concreteness [*t*_(24)_ = 0.434, *p* = 0.668; *t*_(24)_ = 0.154, *p* = 0.879], or arousal [*t*_(24)_ = 1.543, *p* = 0.136; *t*_(24)_ = 1.321, *p* = 0.199] in L1 nor in familiarity [*t*_(24)_ = 1.414, *p* = 0.170; *t*_(24)_ = 0.665, *p* = 0.512], concreteness [*t*_(24)_ = 0.073, *p* = 0.942; *t*_(24)_ = 0.557, *p* = 0.583], or arousal [*t*_(24)_ = 1.558, *p* = 0.132; *t*_(24)_ = 1.590, *p* = 0.125] in L2. Words that co-occur in the same context are semantically related according to the complex network theory (Liu and Cong, 2013). We obtained a context co-occurrence score on WORTSCHATZ^2^ for every prime-target pair. The prime-target pairs of positive words were not significantly different from those of negative words in context co-occurrence in L1 [*t*_(24_) = 1.139, *p* = 0.266] or L2 [*t*_(24)_ = 0.507, *p* = 0.617].

Each pair of prime-target words was used twice: The target word was presented in upper area of the screen one time and in the lower area of the screen one time. To create filler materials, another 26 pairs of positive and another 26 pairs of negative Chinese words were selected, which were similar in familiarity and concreteness to the critical materials. The same number of filler words were determined in English in the same way. The filler words were of middle level in valence (3.93 ± 0.49). The prime and the target word in a filler pair had a context co-occurrence score of zero, which were used in the same way as a critical pair.

The critical materials for Experiment 1 were adapted for use in Experiment 2. The prime in one critical pair was exchanged with the prime in another critical pair. At each treatment level of language by valence, the two words in a trial were in the same language, were of the same valence, but had a context co-occurrence score of zero. Thirteen positive and 13 negative black-white icon-pictures (see Appendix II) were selected from the Internet to be the primes in Experiment 3. Similar to the evaluation procedure of the critical words in Experiment 1, the icon-pictures were evaluated in terms of valence, arousal, and clarity. By clarity we meant to what extent an icon clearly indicated a feeling. The valence evaluation scores of the positive icons (5.55 ± 0.32) were significantly higher than those of the negative icons (2.44 ± 0.24), *t*_(24)_ = 28.004, *p* = 0.000, *d* = 10.996. The positive icons were not significantly different from the negative icons in arousal [*t*_(24)_ = 0.266, *p* = 0.792] or clarity [*t*_(24)_ = 1.559, *p* = 0.132]. Each icon-picture was square shaped and took a space in the center of the screen, having a width of two Chinese characters. To create the materials for Experiment 4, “
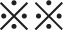
” and “######” were taken as the primes for the Chinese and the English targets, respectively.

#### Procedure

Each of the experiments was conducted in a computer room. The participants were seated in front of the computer screens with their eyes 60 cm horizontally away from the screen centers. A program was designed with DMDX (Forster and Forster, 2003) to present the stimuli and to record the participants' responses. The 15-inch screens had a resolution of 640 × 480 pixels. In each trial, a red fixation-cross “+” remained for 800 ms at the center of the white screen. Then the prime was shown for 287 ms at the screen center. Upon the disappearance of the prime, the target was presented three lines vertically above or below the screen center. The target stayed on the screen for 3000 ms or until a key press was received. The participants were required to press the key “Z” on the keyboard if the target word was positive and to press the key “/” if the target word was negative in meaning. All the string stimuli were presented using the font “Song-36” and the typeface was black. The screen was blank for 1000 ms before the next trial began. Both oral and written instructions were delivered in Chinese and it was emphasized to make responses as quickly and as accurately as possible. There were 16 practice trials followed by 312 experimental trials. The practice trials were randomized for each participant and so were the experimental trials. The participants each received 20 Yuan (3.3 USD) for their participation. The implementation of the experiments was approved by the local government.

### Results

The data were deleted for the trials in which the reaction times were shorter than 200 ms or 3 *SD* above the average and the ratios of the discarded data were 2.3, 1.5 1.8, and 1.8% in Experiments 1–4, respectively. Table 1 displays the results and 2 (language: Chinese or English) × 2 (valence: positive or negative) × 2 (position: upper or lower) by-subject and by-item ANOVAs were done to the data of error rates and reaction times in Experiments 1–4.

**Table 1:** **Participants' error rates (ER) (%) and reaction times (RT) (ms) under the influences of language, valence, and position in Experiments 1–4**.

**Language**	**Valence**	**Position**	**Experiment 1**				**Experiment 2**				**Experiment 3**				**Experiment 4**			
			**ER**		**RT**		**ER**		**RT**		**ER**		**RT**		**ER**		**RT**	
			***M***	***SD***	***M***	***SD***	***M***	***SD***	***M***	***SD***	***M***	***SD***	***M***	***SD***	***M***	***SD***	***M***	***SD***
Chinese	Positive	Upper	1.6	3.3	667	69	1.0	2.7	710	78	0.5	2.0	705	88	0.0	0.0	664	70
		Lower	2.6	4.8	675	83	3.6	7.0	743	85	1.0	2.7	728	90	1.0	2.7	669	77
	Negative	Upper	5.8	6.8	865	104	2.1	4.6	923	87	1.0	2.7	870	100	1.6	3.3	844	88
		Lower	2.1	5.6	807	100	1.0	2.7	874	93	3.6	5.7	850	91	4.7	4.9	843	86
English	Positive	Upper	2.7	4.9	774	79	4.1	5.7	806	69	0.5	2.0	824	109	3.6	5.0	803	52
		Lower	4.5	8.9	759	88	2.6	4.7	799	76	3.7	5.8	820	93	3.6	4.9	755	60
	Negative	Upper	9.3	12.2	968	85	5.8	9.4	1003	93	7.4	10.6	1017	127	6.7	9.6	924	90
		Lower	5.3	7.6	964	112	4.2	5.8	977	89	4.2	7.8	993	117	4.6	7.0	948	88

#### Experiment 1

##### Error rates

**Figure 1:**
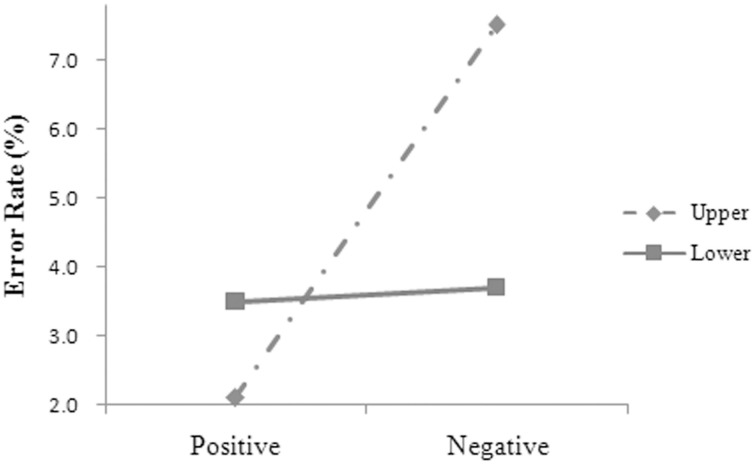
**The two-way interaction between valence and position for the participants' error rates in Experiment 1**.

The main effect was significant for language, *F*_2(1, 48)_ = 5.66, *MSE* = 0.002, *p* = 0.021. The participants' error rates were significantly lower in Chinese (3.0, 0.7%) (*M*, *SE*) than in English (5.3, 0.7%). The main effect was significant for valence, *F*_1(1, 14)_ = 4.58, *MSE* = 0.005, *p* = 0.050; *F*_2(1, 48)_ = 8.57, *MSE* = 0.002, *p* = 0.005, and the two-way interaction was significant between valence and position (see Figure 1), *F*_1(1, 14)_ = 6.41, *MSE* = 0.003, *p* = 0.024; *F*_2(1, 48)_ = 4.65, *MSE* = 0.004, *p* = 0.036. Simple effect analysis showed that the participants' error rates were significantly lower for the positive (2.1 ± 3.6%) than for the negative words (7.5 ± 6.9%) at the upper position, *t*_(14)_ = 2.636, *p* = 0.020, *d* = 1.409, but were not significantly different between the positive (3.5 ± 4.9%) and the negative words (3.7 ± 4.6%) at the lower position, *t*_(14)_ = 0.164, *p* = 0.872.

##### Reaction times

**Figure 2:**
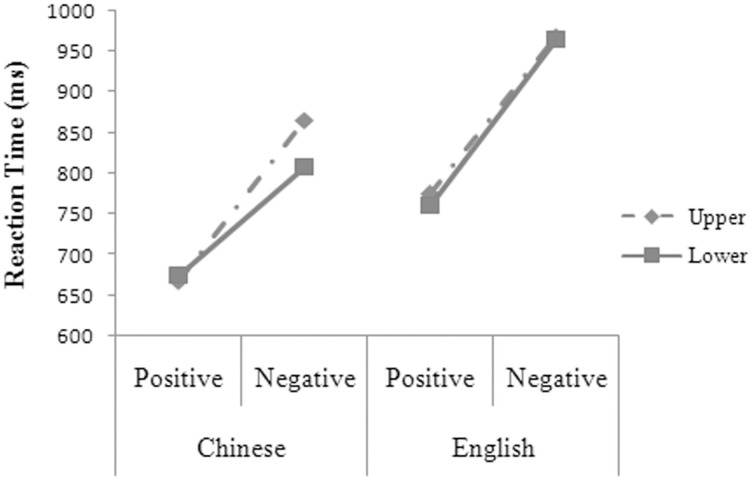
**The three-way interaction between language, valence, and position for the participants' reaction times in Experiment 1**.

The main effects were significant for language, *F*_1(1, 14)_ = 133.59, *MSE* = 2861.87, *p* = 0.000; *F*_2(1, 48)_ = 51.61, *MSE* = 6427.54, *p* = 0.000, and valence, *F*_1(1, 14)_ = 198.25, *MSE* = 5028.25, *p* = 0.000; *F*_2(1, 48)_ = 135.75, *MSE* = 6427.54, *p* = 0.000. The two-way interactions were significant between valence and position, *F*_1(1, 14)_ = 6.52, *MSE* = 2364.37, *p* = 0.023, and between language and valence, *F*_1(1, 14)_ = 4.95, *MSE* = 1810.85, *p* = 0.043. The three-way interaction was significant between language, valence, and position (see Figure 2), *F*_1(1, 14)_ = 6.55, *MSE* = 1671.30, *p* = 0.023. Simple effect analysis suggested that the participants' reaction times were significantly shorter for the positive than for the negative words in Chinese at both the upper [*t*_(14)_ = 10.531, *p* = 0.000, *d* = 5.629] and the lower position [*t*_(14)_ = 7.408, *p* = 0.000, *d* = 3.960]. The participants' reaction times were significantly longer at the upper than at the lower position for the negative words, *t*_(14)_ = 3.435, *p* = 0.004, *d* = 1.836, but were not significantly different at the upper and the lower position for the positive words in Chinese, *t*_(14)_ = 0.633, *p* = 0.537. The participants' reaction times were significantly shorter for the positive (767, 21 ms) than for the negative words in English (966, 24 ms), *F*_1(1, 14)_ = 227.63, *MSE* = 2624.70, *p* = 0.000. The participants had significantly shorter reaction times in Chinese than in English for the positive words at both the upper [*t*_(14)_ = 8.460, *p* = 0.000, *d* = 4.522] and the lower position [*t*_(14)_ = 5.661, *p* = 0.000, *d* = 3.026] and for the negative words at both the upper [*t*_(14)_ = 8.260, *p* = 0.000, *d* = 4.415] and the lower position [*t*_(14)_ = 6.230, *p* = 0.000, *d* = 3.330].

#### Experiment 2

##### Error rates

The main effect was significant for language, *F*_2(1, 48)_ = 4.14, *MSE* = 0.004, *p* = 0.047. The participants' error rates were significantly lower in Chinese (2.0, 0.9%) than in English (4.5, 0.9%).

##### Reaction times

**Figure 3:**
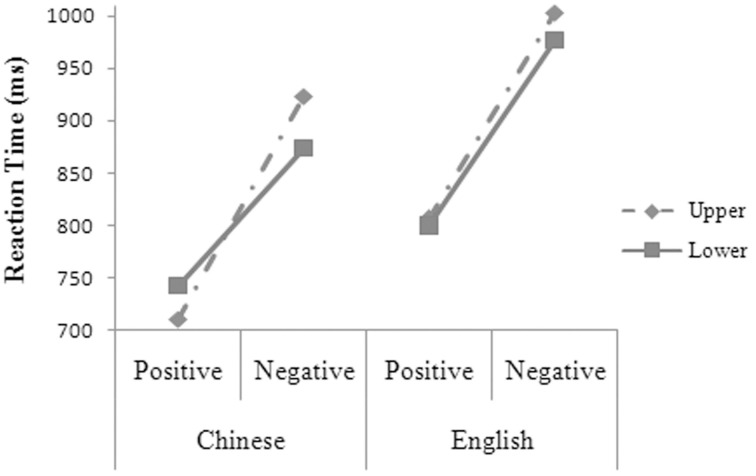
**The three-way interaction between language, valence, and position for the participants' reaction times in Experiment 2**.

The main effects were significant for language, *F*_1(1, 14)_ = 57.95, *MSE* = 3626.75, *p* = 0.000; *F*_2(1, 48)_ = 29.12, *MSE* = 6993.78, *p* = 0.000, and valence, *F*_1(1, 14)_ = 152.59, *MSE* = 6340.46, *p* = 0.000; *F*_2(1, 48)_ = 119.27, *MSE* = 6993.78, *p* = 0.000. The two-way interaction was significant between language and valence, *F*_1(1, 14)_ = 12.41, *MSE* = 1996.86, *p* = 0.003; *F*_2(1, 48)_ = 3.74, *MSE* = 6993.78, *p* = 0.059. The three-way interaction was significant between language, valence, and position (see Figure 3), *F*_1(1, 14)_ = 6.65, *MSE* = 1115.20, *p* = 0.022. Simple effect analysis showed that the participants' reaction times were significantly shorter for the positive than for the negative words at both the upper [*t*_(14)_ = 10.630, *p* = 0.000, *d* = 5.682] and the lower position [*t*_(14)_ = 6.564, *p* = 0.000, *d* = 3.509] in Chinese and at both the upper [*t*_(14)_ = 11.813, *p* = 0.000, *d* = 6.314] and the lower position [*t*_(14)_ = 9.010, *p* = 0.000, *d* = 4.816] in English. In Chinese, the participants had shorter reaction times for the positive words, *t*_(14)_ = 2.905, *p* = 0.012, *d* = 1.553, but had longer reaction times for the negative words, *t*_(14)_ = 4.631, *p* = 0.000, *d* = 2.475, at the upper than at the lower position. The participants' reaction times were significantly shorter in Chinese than in English for the positive words at both the upper, *t*_(14)_ = 6.630, *p* = 0.000, *d* = 3.544, and the lower position, *t*_(14)_ = 3.190, *p* = 0.007, *d* = 1.705, and for the negative words at both the upper, *t*_(14)_ = 6.999, *p* = 0.000, *d* = 3.741, and the lower position, *t*_(14)_ = 6.061, *p* = 0.000, *d* = 3.528.

#### Experiment 3

##### Error rates

The main effect was significant for valence, *F*_1(1, 14)_ = 5.01, *MSE* = 0.004, *p* = 0.042; *F*_2(1, 48)_ = 6.49, *MSE* = 0.003, *p* = 0.014. The participants' error rates were significantly lower for the positive (1.4, 0.4%) than for the negative words (4.1, 1.2%).

##### Reaction times

**Figure 4:**
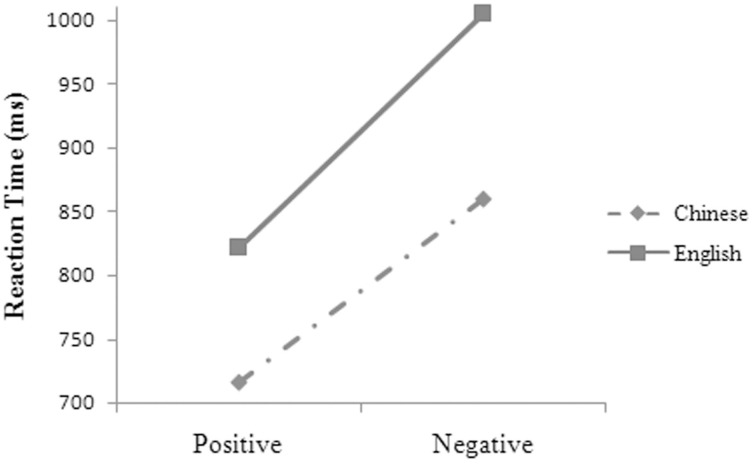
**The two-way interaction between language and valence on the participants' reaction times in Experiment 3**.

**Figure 5:**
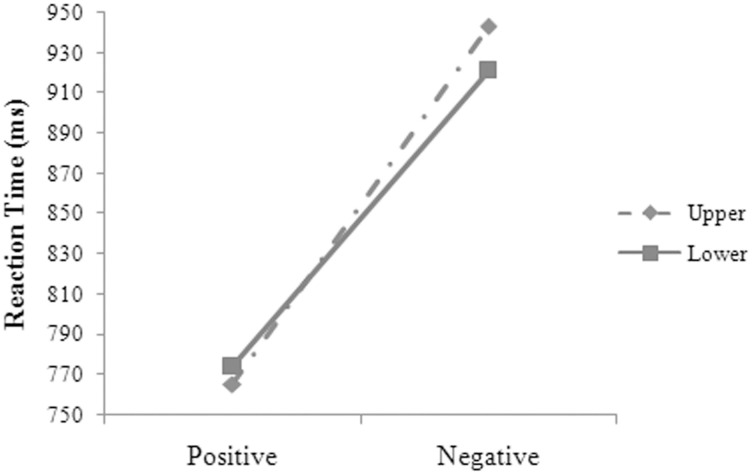
**The two-way interaction between valence and position on the participants' reaction times in Experiment 3**.

The main effects were significant for language, *F*_1(1, 14)_ = 153.70, *MSE* = 3065.30, *p* = 0.000; *F*_2(1, 48)_ = 41.27, *MSE* = 9758.68, *p* = 0.000, and valence, *F*_1(1, 14)_ = 196.63, *MSE* = 4047.84, *p* = 0.000; *F*_2(1, 48)_ = 70.77, *MSE* = 9758.68, *p* = 0.000. The two-way interactions were significant between language and valence (Figure 4), *F*_1(1, 14)_ = 6.94, *MSE* = 1737.22, *p* = 0.020, and between valence and position (Figure 5), *F*_1(1, 14)_ = 5.62, *MSE* = 1304.43, *p* = 0.033. Simple effects analyses suggested that the participants had significantly shorter reaction times in Chinese (717 ± 88 ms; 860 ± 94 ms) than in English (822 ± 95 ms; 1005 ± 119 ms) for both the positive [*t*_(14)_ = 8.811, *p* = 0.000, *d* = 4.710] and the negative words [*t*_(14)_ = 10.916, *p* = 0.000, *d* = 5.835] and had shorter reaction times for the positive than for the negative words in both Chinese [*t*_(13)_ = 13.249, *p* = 0.000, *d* = 7.082] and English [*t*_(13)_ = 11.144, *p* = 0.000, *d* = 6.957]. Their reaction-time differences were significantly larger in English (183 ± 64 ms) than in Chinese (143 ± 42 ms) when valence was changed from positive into negative, *t*_(14)_ = 2.635, *p* = 0.020, *d* = 1.408. The participants' reaction times were significantly shorter for the positive (765 ± 96 ms; 774 ± 86 ms) than for the negative words (943 ± 109 ms; 921 ± 102 ms) at both the upper [*t*_(14)_ = 14.322, *p* = 0.000, *d* = 7.655] and the lower position [*t*_(14)_ = 10.374, *p* = 0.000, *d* = 5.545]. They had significantly longer reaction times at the upper than at the lower position for the negative words, *t*_(14)_ = 2.554, *p* = 0.023, *d* = 1.365.

#### Experiment 4

##### Error rates

The main effects were significant for language, *F*_1(1, 14)_ = 8.70, *MSE* = 0.003, *p* = 0.011; *F*_2(1, 48)_ = 5.58, *MSE* = 0.004, *p* = 0.022, and valence, *F*_1(1, 14)_ = 7.29, *MSE* = 0.002, *p* = 0.017. The participants' error rates were significantly lower in Chinese (1.8, 0.5%) than in English (4.6, 1.0%) and were significantly lower for the positive (2.1, 0.4%) than for the negative words (4.4, 1.0%).

##### Reaction times

**Figure 6:**
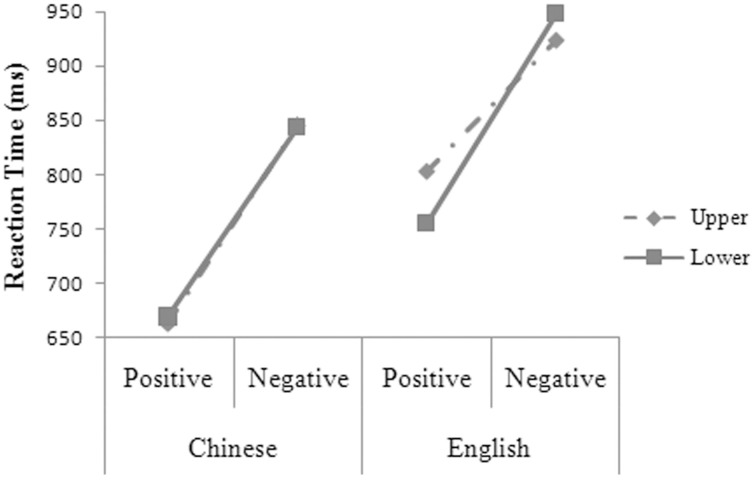
**The three-way interaction between language, valence, and position for the participants' reaction times in Experiment 4**.

The main effects were significant for language, *F*_1(1, 14)_ = 271.75, *MSE* = 1160.93, *p* = 0.000; *F*_2(1, 48)_ = 35.29, *MSE* = 8492.05, *p* = 0.000, and valence, *F*_1(1, 14)_ = 331.76, *MSE* = 2534.91, *p* = 0.000; *F*_2(1, 48)_ = 83.71, *MSE* = 8492.05, *p* = 0.000. The two-way interaction was significant between valence and position, *F*_1(1, 14)_ = 18.84, *MSE* = 428.76, *p* = 0.001. The three-way interaction was significant between language, valence, and position (see Figure 6), *F*_1(1, 14)_ = 19.48, *MSE* = 580.87, *p* = 0.001. Simple effect suggested that the participants' reaction times in Chinese were significantly shorter for the positive (666, 18 ms) than for the negative words (844, 22 ms), *F*_1(1, 14)_ = 621.67, *MSE* = 760.08, *p* = 0.000. The participants' reaction times in English were significantly shorter for the positive than for the negative words at both the upper, *t*_(14)_ = 7.368, *p* = 0.000, *d* = 3.938, and the lower position, *t*_(14)_ = 12.546, *p* = 0.000, *d* = 6.706. Their reaction times in English were significantly longer at the upper than at the lower position for the positive words, *t*_(14)_ = 4.051, *p* = 0.001, *d* = 2.165, but were significantly shorter at the upper than at the lower position for the negative words, *t*_(14)_ = 3.629, *p* = 0.003, *d* = 1.940. The participants had significantly shorter reaction times in Chinese than in English for the positive words at both the upper, *t*_(14)_ = 10.133, *p* = 0.000, *d* = 5.416, and the lower position, *t*_(14)_ = 6.159, *p* = 0.000, *d* = 3.292, and for the negative words at both the upper, *t*_(14)_ = 7.634, *p* = 0.000, *d* = 4.081, and the lower position, *t*_(14)_ = 8.566, *p* = 0.000, *d* = 4.579.

## General discussion

With the same set of affective words as the targets, which were presented in the upper or the lower part of the screen, we did four affective priming experiments, in each of which language (L1 or L2), valence (positive or negative), and position (upper or lower) were variables. The primes, presented at the center of the screen, were affective words in Experiments 1 and 2, affective icon-pictures in Experiment 3, and neutral strings in Experiment 4. In the same language as the targets, the primes were semantically related to the targets in Experiment 1 but were not semantically related to the targets in Experiment 2. The affective primes and the targets were congruent in valence in the first three experiments. Fifteen unbalanced bilinguals were required to make valence judgments on the targets in each experiment, with their error rates and reaction times recorded.

The participants' performance was generally poorer in L2 than in L1. They found it more difficult to recognize affective words in L2 than in L1, a finding which is in agreement with the RHM and the sense model. Most importantly, their performance reflected different patterns of interaction between the variables across the experiments.

### Metaphorical association

In Experiment 1, the participants had longer reaction times and higher error rates for the negative targets in L1 at the upper than at the lower part of the screen, but their performance was immune to the influence of position in L2. In Experiment 2, the participants' reaction times were longer for the negative words and were shorter for the positive words in L1 at the upper than at the lower part of the screen but were not influenced by position in L2. In Experiment 3, the participants had longer reaction times at the upper than at the lower part of the screen for the negative targets, regardless of whether the targets were in L1 or L2. In Experiment 4, no metaphorical association was indicated between affect and position.

That is, metaphorical association between affect and vertical position (Meier and Robinson, 2004) was revealed in error rates as well as in reaction times on the negative words in L1 in Experiment 1 and were revealed in reaction times on both the positive and the negative words in L1 in Experiment 2. The differences between Experiment 1 and 2 in the influence of position on the participants' performance in L1 must be because of the strength differences of semantic relations between the primes and the targets across the two experiments. Further investigation is needed into the interaction of the prime-target semantic relations and the targets' presentation positions, but both Experiments 1 and 2 yielded strong evidence in support for metaphorical association between affect and vertical position (Meier and Robinson, 2004) in L1 but not in L2.

The influence of valence of affective words is repeatedly indicated on participants' performance in cognitive studies (Ferré et al., 2011), and it is believed that there are reciprocal interactions between affective and cognitive processing (Okon-Singer et al., 2013) in recognizing an affective word. By affective processing we mean “somatovisceral responses triggered by automatic appraisal” (Pavlenko, 2012, p. 409) in this process. According to Pavlenko (2012), two lines of automatic processing, semantic and affective processing, coexist in detecting an affective word. The participants must have automatically processed the affective as well as the semantic contents of the L1-affective-word primes in Experiments 1 and 2. Consistent with Meier and Robinson (2004), it was the affective processing that was responsible for the observed metaphorical associations between affect and vertical position in L1. However, the participants might not be able to process the prime words in L2 in an effective way and thus were unable to have their performance influenced by the L2 targets' positions. Similarly, the participants were likely to be more sensitive to perceive the targets in the lower than in the upper part of the screen as the result of affective processing of the negative icon-picture primes in Experiment 3. Indeed, their reaction times did change in agreement with the metaphorical association between affect and vertical position, regardless of the targets' presentation language.

Furthermore, that metaphorical association between affect and vertical position was separate from semantic processing of affective words in L1 in the present study is similar to Ferré and Sánchez-Casas's (2014) separation of semantic and affective aspects of L1 words in a lexical decision task.

### Interactions between language, valence, and position

Semantic representations develop as the result of humans' direct interactions with the physical world in combination with their uses of language (Andrews et al., 2009). When learning a word through direct experiences with the physical world, one develops semantic representations that are based on sensory perceptions. In language practices a human experiences words which help inspire and thus are associated with his or her perceptual memories. His or her representations for abstract words that are acquired solely through language uses are grounded in cognition, probably mainly through physical metaphor (Lakoff and Johnson, 1999). As far as affective words are concerned, “mental states like emotions are constructed from the dynamic interaction of physiological states, situation-specific information, and conceptual knowledge” (Caldwell-Harris, 2014, p. 1).

However, the case might be different with L2 words in unbalanced bilinguals, who mainly enlarge their L2 vocabulary not by means of using the language. An unbalanced bilingual such as the participants of the present study usually begins to learn an L2 word (E_x_) by memorizing its L1 translation equivalent (C_x_) and mapping it onto the meaning(s) of C_x_ as listed in the glossary book(s), which is of a typical manner of English learning in non-English-majored college students in mainland China (Li et al., 2011). Actually, memorization is a recognized strategy in English learning and many studies were conducted on how to train English learners' memory strategies (e.g., Fang and Shao, 1996; Fan et al., 2008). After using rote memory and gaining limited experiences with the word in textbook(s), he or she learns E_x_ and develops certain semantic representations, which overlap with some of the semantic representations for C_x_. The strength of association between E_x_ and the semantic representations increases as the bilingual increases his or her familiarity with E_x_ in his or her textbook learning. Unless he or she tries to get more familiar with E_x_ by means of meaningful practice in the language, such as extended reading or living in the country of the target language, the number of representations that are accessible for E_x_ are not likely to change in size. In other words, only in using the L2 translation for an affective word in L1 in emotional experiences can a bilingual develop his or her emotional aspect of representation that can be automatically activated in L2.

“Good” and “bad” are two of the limited number of universal semantic primes (Goddard and Wierzbicka, 2002) that are “intelligible for anyone both within and across languages and cultures” (Wierzbicka, 2005, p. 583). The semantic representations for E_x_ overlap with some of those for C_x_ that are universal in nature. Metaphorical association between affect and vertical position (Meier and Robinson, 2009) is an indication of the emotional aspects of affective words that are grounded in cognition (Lakoff and Johnson, 1999). Many aspects of representations for C_x_ that are grounded in cognition are not accessible for E_x_ for bilinguals such as the participants of the present study. Therefore, the participants had the same pattern of poorer performance on the negative than on the positive words in L2 as they had in L1 in the present study, suggesting the overlap of semantic representations between L1 and L2 that are universal in nature. However, they failed to activate metaphorical association between affect and vertical position when perceiving the L2 primes, indicating their lack of representations for the emotional content of the affective words. This is in agreement with Dewaele (2004) that affective words are intuitively more arousing in emotion in L1 than in L2, especially for late bilinguals (Altarriba, 2008). Similarly, Li et al. (2011) indicated that unbalanced bilinguals had a symmetric pattern of relative awareness of taxonomic and thematic associations among concrete words in L1, in agreement with Lin and Murphy (2001), but had an asymmetric pattern of relative awareness of taxonomic and thematic associations among concrete words in L2. Li et al. (2011) argued that unbalanced bilinguals were weak at thematic associations among concrete words because of their lack of practices in L2.

Our proposal regarding unbalanced bilinguals' semantic representations for affective words in L1 and L2 is consistent with the previous theories (Potter et al., 1984; De Groot, 1992; Kroll and Stewart, 1994; Finkbeiner et al., 2004), but the common semantic representations for L1 and L2 lexicons as indicated in these theories appear to refer to those that are universal in nature. Many of the semantic representations for L1 words that are not accessible by the L2 translation equivalents as indicated by Finkbeiner et al. (2004) must be cognition-grounded in nature. Of course, those who learn L2 words through L2 practices as well as with the help of L1 translation equivalents are not only able to gain access to their semantic representations that are universal in nature but also are likely to develop some associations between their perceptual memories and the L2 words. The theoretical implication of the present study is that word types should be considered in the development of models on bilinguals' representations for L2 words. As far as affective words are concerned, for example, both the emotional and the semantic contents should be taken into consideration in improving the existing models.

Why the participants performed more poorly on the negative than on the positive targets can be explained as follows. It is repeatedly shown that more resources are assigned to processing negative stimuli than processing positive stimuli in cognitive tasks (Kiken and Shook, 2011). Moreover, Unkelbach et al. (2008) proposed the density hypothesis which assumes that “positive information is more similar to other positive information, in comparison with the similarity of negative information to other negative information” (p. 36). The result of a processing advantage of positive over negative information in the present study just confirms this theoretical proposition.

As to why the participants had significantly longer reaction times for the positive and shorter reaction times for the negative targets in L2 at the upper than at the lower position in Experiment 4, we think that it is because of attention. In this experiment no priming effect was expected. The participants' responses only reflected their perception of the targets. It is comfortable to work on the computer with the screen more than 10° below the eye level (Jaschinski et al., 1998; Wimalasundera, 2006). Visual perception seems more likely to cause eyestrain (Jaschinski et al., 1998) and to cause pain in the neck and shoulder (Wimalasundera, 2006) when the screen is higher than 10° below the eye level. Thus, it might have been physically more demanding for the participants in the present study to attend to a word at the upper than at the lower part of the screen. This difference was not revealed in the participants' performance in L1 because the L1 targets were easy to perceive. However, the difference in physical effort to attend to the L2 targets in the upper and the lower area of the screen did seem salient enough to be reflected in the participants' reaction times. After all, more cognitive resources are needed for words in L2 than in L1. The participants' longer reaction times for the positive targets in L2 at the upper than at the lower position must be an indication of their assignment of more physical effort to attend to the positive targets in L2 in the upper than in the lower position on the screen. They had longer reaction times for the negative L2 targets at the lower than at the upper position because of their inclination to be alert to the negative targets in L2 was compatible with the need for physical effort to attend to stimuli at the upper position but was incompatible with the need for physical effort to attend to stimuli at the lower position. This asymmetric pattern of demand for attention resources was not observed in the first three experiments because the participants' perception-oriented cognitive resources had been consumed by metaphorical associations between affect and vertical position in automatically perceiving the affective primes. Of course, studies are needed to provide more direct evidence in support of the argument that unbalanced bilinguals' attention is more likely to be influenced in L2 than in L1.

In conclusion, semantic representations for affective words can be shared by L1 and L2 lexicons, but metaphorical association between affect and vertical position works in L1 but not in L2 for unbalanced bilinguals such as the participants of the present study. The implication is two-fold. Theoretically, the existing theories on bilinguals' representations for L2 words should be improved with emotional aspects of affective words taken into consideration. Practically, learners should learn L2 by using the language. Otherwise, they are at the risk of not being able to develop semantic representations for L2 words that are grounded in cognition.

### Conflict of interest statement

The authors declare that the research was conducted in the absence of any commercial or financial relationships that could be construed as a potential conflict of interest.

## Figures and Tables

**Figure A1:**
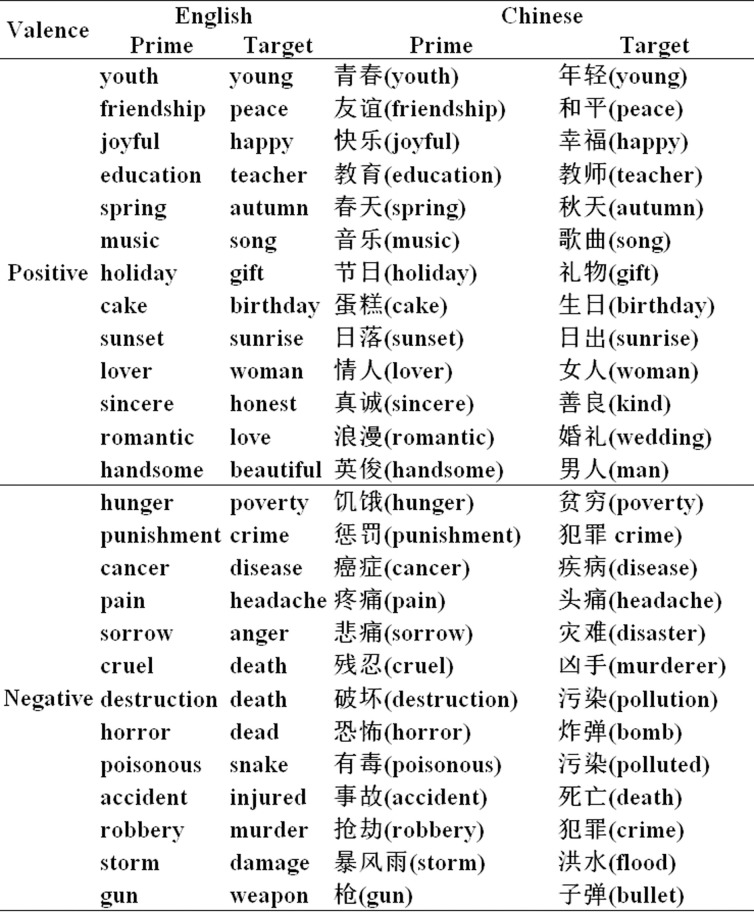
**The stimuli at each treatment level of language by valence in Experiment 1**.

**Figure A2:**
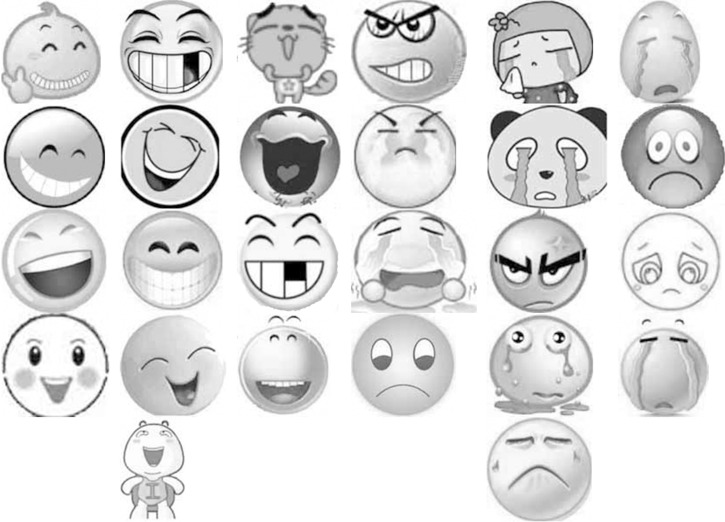
**Positive and negative icon-pictures in Experiment 3**.

## Footnotes

^1^The CI for the effect sizes in the *t*-tests was 95%.

^2^WORTSCHATZ. University of Leipzig, http://corpora.uni-leipzig.de.
